# Computational Binding Study Hints at Ecdysone 20-Mono-Oxygenase as the Hitherto Unknown Target for Ring C-Seco Limonoid-Type Insecticides

**DOI:** 10.3390/molecules29071628

**Published:** 2024-04-05

**Authors:** Ramsés E. Ramírez, Ricardo E. Buendia-Corona, Ivonne Pérez-Xochipa, Thomas Scior

**Affiliations:** 1 Departamento de Fisicomatemáticas, Facultad de Ciencias Químicas Benemérita, Universidad Autónoma de Puebla, Prol. 24 Sur, Puebla 72570, Mexico; ramses.ramirez@correo.buap.mx (R.E.R.); ricardo.buendia96@gmail.com (R.E.B.-C.); 2 Departamento de Bioquímica Alimentos, Facultad de Ciencias Químicas Benemérita, Universidad Autónoma de Puebla, Prol. 24 Sur, Puebla 72570, Mexico; ivonne.perez@correo.buap.mx; 3 Laboratorio de Simulaciones Moleculares Computacionales, Facultad de Ciencias Químicas Benemérita, Universidad Autónoma de Puebla, Prol. 24 Sur, Puebla 72570, Mexico

**Keywords:** ecdysone 20-monooxygenase, ecdysone, C-seco limonoid, insecticide, molecular mechanism, docking

## Abstract

The insecticidal property of ring C-seco limonoids has been discovered empirically and the target protein identified, but, to date, the molecular mechanism of action has not been described at the atomic scale. We elucidate on computational grounds whether nine C-seco limonoids present sufficiently high affinity to bind specifically with the putative target enzyme of the insects (ecdysone 20-monooxygenase). To this end, 3D models of ligands and the receptor target were generated and their interaction energies estimated by docking simulations. As a proof of concept, the tetrahydro-isoquinolinyl propenamide derivative QHC is the reference ligand bound to aldosterone synthase in the complex with PDB entry 4ZGX. It served as the 3D template for target modeling via homology. QHC was successfully docked back to its crystal pose in a one-digit nanomolar range. The reported experimental binding affinities span over the nanomolar to lower micromolar range. All nine limonoids were found with strong affinities in the range of −9 < ΔG < −13 kcal/mol. The molt hormone ecdysone showed a comparable ΔG energy of −12 kcal/mol, whereas −11 kcal/mol was the back docking result for the liganded crystal 4ZGX. In conclusion, the nine C-seco limonoids were strong binders on theoretical grounds in an activity range between a ten-fold lower to a ten-fold higher concentration level than insecticide ecdysone with its known target receptor. The comparable or even stronger binding hints at ecdysone 20-monooxygenase as their target biomolecule. Our assumption, however, is in need of future experimental confirmation before conclusions with certainty can be drawn about the true molecular mechanism of action for the C-seco limonoids under scrutiny.

## 1. Introduction

With the human population constantly growing, a need has arisen for the industrialization of agriculture and food production. Both cannot undergo endless optimization procedures without the use of herbicides or pesticides. The cultivation of food plants has to be conducted in new ways, so new forms of field care like insecticides can be developed. One of these alternatives is so-called “biopesticides”, which constitute natural compounds obtained from living organisms that contribute to the elimination of pests. Their advantage is observed in the specificity they have, with only pests affected by the compound [1].

Recently, publications have reported the biological activity of plant compounds called “limonoids” [2,3,4,5,6,7,8,9,10,11], emphasizing their high insecticidal potency as biopesticides at the macro- and microscopic levels, which gives us the opportunity to study their possible mechanism of action at the molecular level. We assume the following mechanisms of action for the limonoid compounds in our theoretical study: Upon binding to their target receptor, the protein ecdysone 20-monooxygenase (E20MO for short), the limonoid ligands interrupt the downstream release of ecdysteroid hormones. As a direct consequence, certain biological functions of the living insects are hampered at different levels, consequently causing their death [7,10]. The hitherto unknown affinities (binding energies) to the E20MO receptor can be estimated on computational grounds. So-called ligand–receptor docking studies can be carried out to simulate the molecular interaction of the limonoid ligands against their putative target protein, E20MO, which was postulated as a target prior to this study [10] and cited by [11]. In acro-chemistry, as in medicinal chemistry, computing physicochemical properties or simulating biochemical reaction pathways helps us to understand the action mechanism of drugs or chemical substances like the limonoids in our in silico study. In addition, awareness about the use of biopesticides can be spread that may arise after molecular characterization for application in everyday life. Limonoid agents of type “C-seco” (acronym: LACS) belong to the secondary metabolite class of terpenoids, which can be isolated from the roots of *Azadirachta indica*, A. Juss. This Indian plant is regionally better known as “Neem tree” and belongs to the Meliaceae plant family, which in turn branches into more than 50 genera and more than 1400 species dispersed in tropical and sub-tropical climates throughout the world.

## 2. Results

**Figure 1:**
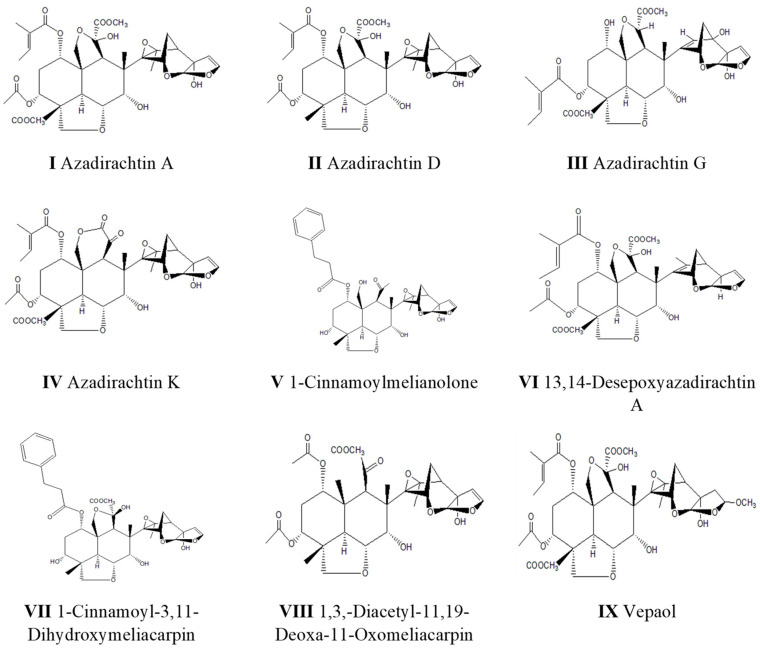
Structures of the nine ring C-seco limonoids and their identity labels.

**Figure 2:**
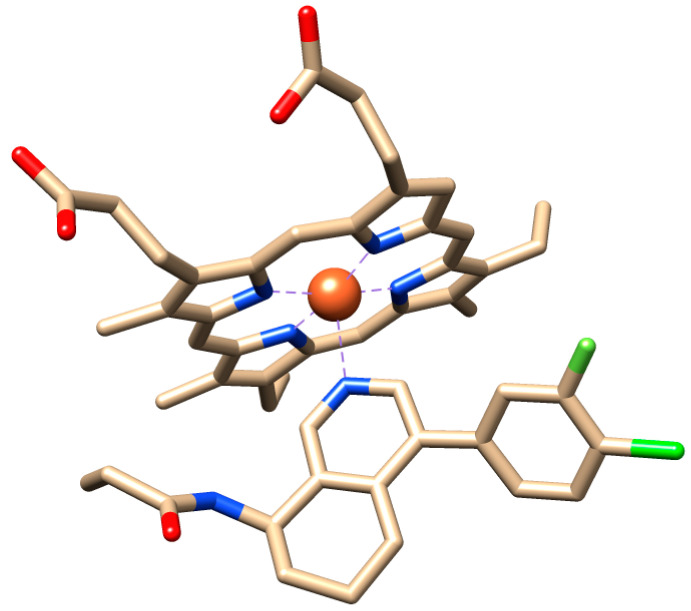
Display of the liganded heme group at the binding site of reference crystal structure by X-ray diffraction at 3 Å resolution from RCSB Protein Data Bank [13]. The complex represents aldosterone synthase (CYP11B2) with a bound tetrahydro-isoquinolinyl propenamide derivative. Its protein backbone served as a 3D template for target protein modeling by homology. In addition, it was used as a reference for docking validation by (successful) back docking of its reference ligand QHC into its experimentally determined pose in the aldosterone synthase complex (CYP11B2). In addition, the computed value is in excellent keeping with the experimental value range of affinities. Color code for atoms of stick models: oxygen atoms O in red, N in blue, C in beige and halogen atoms F and Cl in green. H atoms omitted and central iron atom in complex as red ball.

Stable structures with local energy minima in the ground state were generated for all 3D models. A comparison of azadirachtin A (Aza) against the optimized structure was carried out using a nuclear magnetic resonance Mosher approach [5]. This structure served as a scaffold for constructing the first limonoid Aza to optimize it and to obtain its ground-state structure (see Figures S1–S10 in Supplementary Information). Then, by adding the substituents and optimizing under the B3LYP protocol with the 6-311+G** basis set, the eight remaining C-seco limonoid structures were obtained as a valuable asset; the steroid scaffold of ecdysone was found in the quantum chemistry software Gaussian 16 compound repository [12]. Again, the missing substituents were added, and the complete molecule was optimized under B3LYP/6-311+G** (see Figure 1). In Figure 2 the reference ligand ecdysone is shown in its active conformation attached to a heme group at the putative target binding site.

### 2.1. Target Modeling by Primary Sequence Homology

No PDB entry was found in the RCSB Protein Data Bank (https://www.rcsb.org/, accessed on 10 November 2023 [13]) for target Ecdysone 20-monooxygenase (EC: 1.14.99.22), AKA under its short name E20MO or alternative names CYPCCCXIVA1 or mitochondrial cytochrome P450 314a1 (CYP314A1). The protein sequence of the target enzyme E20MO was obtained from the Uniprot database (Q9VUF8) for the fruit fly (Drosophila melanogaster) prior to the BLASTp search for related 3D templates to model E20MO by homology against the Protein Data Bank (https://blast.ncbi.nlm.nih.gov/Blast.cgi?PAGE=Proteins, accessed on 19 November 2023) [14]. In the next step, eight Protein Data Bank entries were preselected and retrieved (see Table S1 in Supplementary Information). Since no structure totally matched our target protein, our selection criteria were as follows: (i) high identity percentage, (ii) similar biological activity (enzyme class, oxidation by heme group) as well as (iii) chemical similarities between PDB ligands and our limonoids.

Prior to selecting the final template, an additional PDB search was carried out looking for ligands with chemical similarities to limonoids or the insect hormone ecdysone (see Table S2 in Supplementary Information). The idea was to find ligand–receptor interaction patterns at the binding sites of protein complexes with ligands that were closely related to our limonoids.

**Figure 3:**
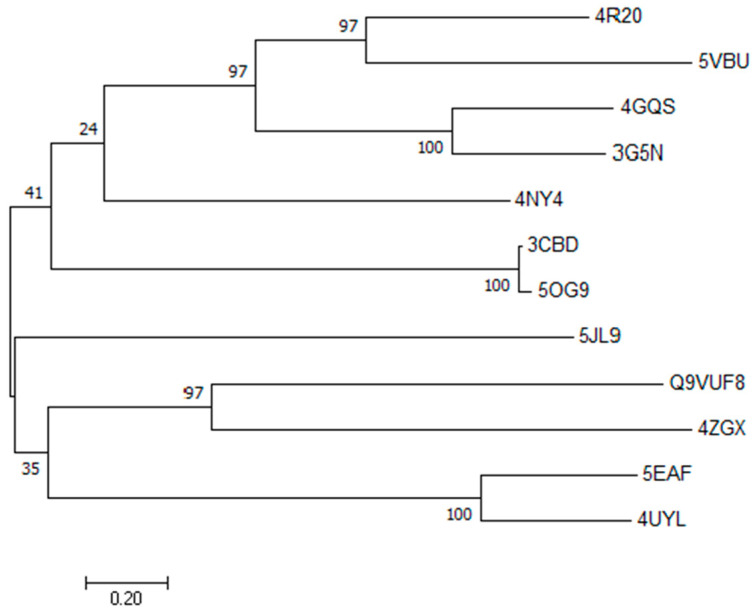
Phylogenetic tree diagram for potential 3D templates to model the target structure. The rightmost labels display the PDB entry codes (see Supplementary Information Tables S1 and S2). Label Q9VUF8 is the query sequence to relate with the PDB sequences. Since PDB entry 4ZGX is only one branching point apart from Q9VUF8 it was selected as the 3D template. It is followed by two preselected proteins (4UYL and 5EAF). All others are far more distant. The numbers indicate successful bootstrapping cycles to obtain the branching points as shown. Phylogeny reconstruction was achieved under MEGA 7 [26].

Another criterion was to assess the evolutionary distance in a phylogenetic tree. It was based on the primary sequences of our template candidates (see Supplementary Information Table S1 [15,16,17,18,19,20,21,22] as well as Table S2 [23,24,25]). A plethora of heme-containing PDB entries exist. So, there was a need to focus on the eight preselected template candidates, which are all closely related proteins by homology. The phylogenetic tree analysis was carried out in the MEGA 7 software [26]. It can display the protein relatedness in a diagram (Figure 3). The branches (ramifications) of the tree along with the line length reflect the evolutionary distance between 3D template candidates (rightmost labels in Figure 3 are the PDB entries) and target protein (leftmost starting line or branch in Figure 3). As a direct result, only a few PDB entries had to be retrieved for inspection of their 3D structures for potential use as 3D templates for target protein model generation by homology. Statistics was applied with 1000 bootstrapping cycles [27] for a Jones–Taylor–Thornton approach [28] (see Figure 3).

Since only the primary structure of the insect target enzyme E20MO is known to date (Uniprot id: Q9VUF8, [14]), a highly suited 3D template was chosen for target protein modeling thanks to several observations: (i) The target constitutes a heme-bearing enzyme belonging to the oxydoreductase family (EC 1.14.99.22), while the selected template protein (PDB entry 4ZGX [18]) also belongs to the oxydoreductase enzyme class (EC 1.14.15.5). The template is the human heme-bearing cytochrome P450 Cyp11B2—AKA aldosterone synthase. (ii) Its crystal structure was completely resolved, and (iii) it is not only structurally related to the target but also functionally because it catalyzes the hydroxylation step of steroid substrates in the biosynthesis of the mineralocorticoid aldosterone. 

**Figure 4:**
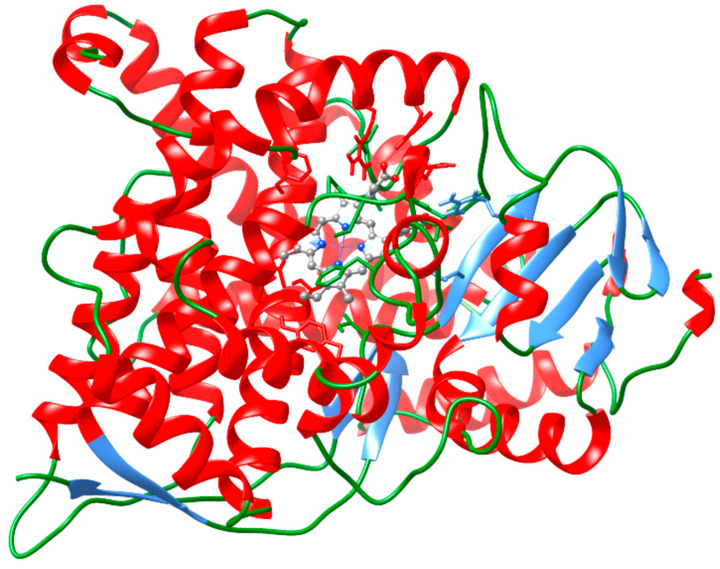
Display of the 3D model E20MO4ZGX. It was generated on the backbone coordinates of 3D template 4ZGX as input under Swiss-model software [29]. At the center of its active site is the heme group for redox catalysis. The cavity remains unoccupied until docking is performed. Backbone ribbon colors: in red helices; in blue beta strands; in green loops. The heme group and adjacent residues are represented in ball and stick display. Atom colors: in cyan C, in red O, in blue N atoms. H atoms were undisplayed. Visualization using software USCF Chimera 1.16 [34].

The target homology model was generated based on the Cartesian coordinates of 4ZGX using the Swiss-model software [29] (see Table S1 in Supplemental Information). It was labeled as E20MO4ZGX (see Figure 4). After the potential energy minimization, the final target crystal complex differs in its overall geometry from the template. The differences were measured as the root mean square deviation of positions between atom pairs (RMSD = 3 Å) In the next step, we added the heme group of the CYP P450 enzymes under Swiss-PDB-Viewer, applying its superposition tool Magic Fit [30]. The binding cavities were inspected for possible limonoid binding and the unoccupied volumes at the heme site measured. The unoccupied cavity for E20MO4ZGX is 250 Å^3^. 

Moreover, the template protein constituted the best choice with regard to structural fitness and chemical propensities to accommodate ligands (molecular weight, element formulae, volume, terpenoid likeness, etc.). The large difference in binding site volume is well known for the CYP P450 enzyme family [31,32]. It undergoes induced fit processes to adopt new conformations, which enable them to accommodate a wider range of substrates for oxidation or hydroxylation reactions [33]. In the following procedure, docking showed that target model E20MO4ZGX was able to accommodate even the largest ligands. In the case of 4ZGX, experimental binding data are available, which can be exploited to evaluate whether the software is capable of docking the ligand back into its binding site with the observed binding mode and pose (EC50: 7 nM–31.4 μM) [18].

### 2.2. Molecular Simulation Details

With the 3D models of limonoid ligands and receptors at hand, the affinities were assessed through docking simulations (see Table S5 in Supplementary Information). To compare and validate the computed results, we re-docked crystal structures with ligands chemically related to ecdysone and limonoids too. The binding energy results were evaluated on a logarithmic scale to evaluate the potential affinities between limonoids and the target protein and confirm the insecticidal action mechanism at a molecular level. Thanks to the back docking studies, the reliability of our blind docking limonoids to target is fairly enhanced since the successful back docking demonstrates that molecules similar in shape and chemistry can also be expected to perform well. Concerning the chemical relatedness, prior to docking, 841 PDB hits (as of 14 November 2018) were screened for chemically related ligands in a complex with the heme-bearing enzyme by a search pattern for carbon, hydrogen and oxygen-containing small organic compounds (formula “CxHyOz”). The presence of aliphatic rings and/or terpenoid-like complexity was also searched (see Table S3 in Supplementary Information) [35,36,37,38,39,40]. As a direct result, it was inferred that high affinity between the limonoids and E20MO could be expected.

**Figure 5:**
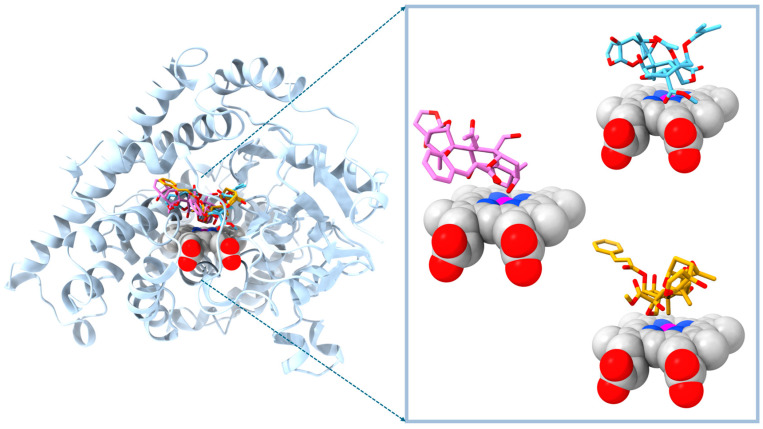
Display of target model E20MO with the docking poses of the three strongest binders in superposition. **Left side panel**: ribbon display of the backbone of ecdysone 20-mono-oxygenase target in steel blue. Space fill model of the active site heme group (bottom center). **Richt side panel**: magnified view on the liganded heme group with docked poses of compound IV (top). compound V (center) and compound VII (bottom). Hydrogen atoms omitted. Atom color code: red O, blue N, carbon atoms of compound IV, V or VI in sky blue, magenta or goldenrod, respectively. Model generated on docking output spatial coordinates by molecular modeling software UCSF Chimera 1.16 [34].

The different types of chemical similarities between complex ligands and target ligands (limonoids) were analyzed and documented (see Tables S1–S4 in Supplementary Information). The ligands comprise the nine limonoids I-IX as well as the insect hormone ecdysone and the ligand QHC, which was extracted from its crystal structure and constitutes the 3D template (4ZGX). The binding values were obtained by blind docking for I–IX and reference ecdysone (X) against target model E20MO4ZGX. The reference ligand QHC was blind docked against target model E20MO4ZGX and back docked into the active site of its crystal structure 4ZGX [18]. In total, twelve docking studies were carried out under the same program settings. At this stage, our docking simulations estimated the affinities to target E20MO (4ZGX). The computed numeric results were compared at a logarithmic scale (see Table S5 and Figure 5). The literature attests that ligand QHC (*N*-[(8*R*)-4-(4-chloro-3-fluorophenyl)-5,6,7,8-tetrahydroisoquinolin-8-yl]propanamide) of 4ZGX (see Table S5 in Supplementary Information) binds with binding affinities in a nanomolar range between 7 and 31 400 (31.4 µM) [18]. Our results in Table S5 show a K_i_ value of 4 nM for the back docked QHC at the binding site of its crystal structure. This result lies in good keeping with the aforementioned affinity range (7 to 31 400 nM). Back docking validates our docking approach in a twofold manner: (i) the docked pose of reference ligand QHC, which is close to the observed X-ray pose; and (ii) the computed affinity value close to the experimental range. The Gibbs-free energy of binding (ΔG) can be converted into molar concentrations by the following thermodynamic equation: ΔG = R × T × ln(K/C), where R is the universal gas constant, K the inhibition constant at equilibrium as well as T the temperature on the absolute Kelvin scale. As a crude approximation for this conversion, any difference in ΔG between two ligands corresponds approximately to a tenfold difference in their inhibitory constants, which is measured in molarity units, i.e., expressed as the wanted concentration. The molar inhibition constant values of the final poses reflected either equal or 10- to 100-fold lower binding strength of limonoids than the values obtained by back docking of the chemically related ligand QHC. Three ligands performed best, i.e., equal concentrations or amounts in comparable order were required to yield the same inhibition effect with the validated reference ligand QHC. The three were compounds **IV**, **V**, and **VII**. A tenfold higher concentration was required, i.e., the compounds acted in a tenfold lower order, namely compounds **II**, **III**, **VI**, **VIII,** and **X**. The latter is ecdysone, itself. A third group had 100-fold weaker affinity, i.e., they needed 10- to 100-times higher concentrations to cause the same enzyme-blocking response. These compounds were **I** and **IX** (see Table S5 Supplementary Information). 

All ligands were displayed in the superposition of their final docked poses for eye-sight verification (see Figure 5). Moreover, the figures of individual superpositions of ligands to the target were also documented (see Figures S1–S10 in Supplementary Information).

**Figure 6:**
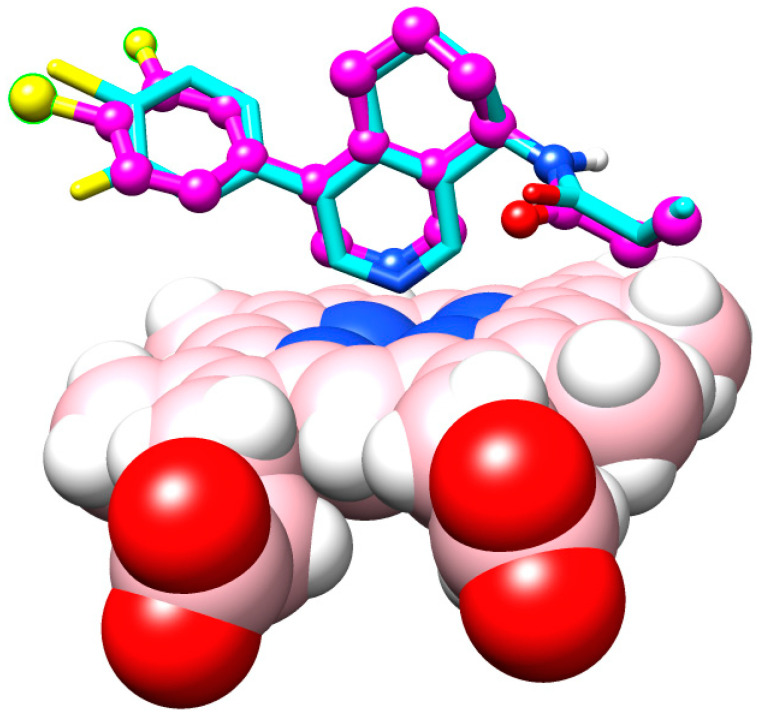
Display of the backed docked reference ligand in superposition with its crystal pose. At the binding site only, both poses of the ligand QHC above the heme group were displayed. The central iron cation is buried in the center below thee binding nitrogen atom of the ligand. The computed pose is the most populated cluster of solutions which shows the second strongest binding affinities of all 256 predicted docking solutions. Of note the computed phenyl ring conformation is flipped around its binding axis to the scaffold by 180 degrees (leftmost ring on both ligands). The underlying bond formally constitutes a single bond but is bridging the resonance between two aromatic rings (pi electron conjugation). Since docking is based on molecular mechanics were partial charges and polarizations can only be presented by electrostatic nature, a trade-off has to be made between two options: to (i) either “freeze in” the original torsion angle between F-,Cl-phenyl ring and its scaffold aromatic ring, or (ii) define it as rotatable bond prior to docking. For unbiased validation random start position and free rotation was chosen. Color code: yellow F and Cl, magenta C- atoms of ball and stick model by docking, light blue C-atoms of stick model from 4ZG. Space fill model: heme group. Atom colors: N blue, red O, pink C, white H. All hydrogen atoms on ligand were omitted. Molecular modeling software UCSF Chimera 1.16 [34].

If ligands and receptors are similar, any successful back docking supports the blind docking simulations (see Figure 6). This condition was already taken into account by one of the three selection criteria, which was to look not only for related proteins but also for similar ligands (see Section 2.1). For target E20MO4ZGX, a threefold final validation step was carried out. We reproduced the final poses and affinities of experimentally known crystal complexes, which we took from our data collection (see Tables S1–S4). To this end, three reported enzyme–ligand crystal structures were successfully docked back: (i) 4ZGX with its ligand “QHC”; (ii) 3SN5 with its ligand “cholest-4-en-3-one”; and (iii) 5FOI with its “micinamycin” ligand (see Table S4). In addition, results were found in keeping with computed scoring data for azadirachtin and 1-cinnamoylmelianolone (Limonoid **V**). 

## 3. Discussion

Although the proposed biomolecular target has never been elucidated by experimental evidence, this study sheds light on the theoretical molecular mechanism of action of natural limonoids for the putative target protein ecdysone 20-mono-oxygenase. Our findings are of a preliminary character, but the research wealth lies in our proposal to guide future research towards this putative target [7,10]. New studies could include experimental assays to identify the target biomolecule, as was the case with anti-melanogenic limonoids [41]. In an optimal situation, for plant extractions and the analytical identification of limonoids, their partial or total synthesis of new derivatives after molecular design and biological activity assays could be combined, as reported in a study for berberine chloride [42]. Limonoids, as other essential oil ingredients, possess a vast applicability range for industrial food production as well as industrial agriculture to developed sustainable agrochemicals, all of which lends them a dual function as a nontoxic food additive for humans and as insecticide agents [6,43]. Their potential assets have been outlined in the literature recently [44,45]. In particular, in the context of environmental toxicity, biodegradable limonoids are less harmful than older synthetic pesticides, which persist as pesticide residues in crops in fields or are washed into surface water and end up contaminating drinking water or food plants and fruits, all of which can be detected in pesticide residue analyses [46,47]. 

## 4. Materials and Methods

The three-dimensional structures of our studied LACS were generated applying ab initio optimization of their geometries, solving the Schrödinger equation by the approximation of atomic orbitals. Electronic charge calculations were performed using the RHF method with an STO-3G base to explore atomic reactivities in the molecules (see Figure 1). We optimized the molecular structures of our limonoids and reference molecule ecdysone from methods called ab initio using the Gaussian 16 & GaussView6 package [12]. In a subsequent step, Aza was chosen as a general scaffold to formulate the Z matrices under the ChemSpider Web page (job ID: 4444685) for later use as input data for Gaussian 16 [48]. Precisely, the Z-matrix of Aza was automatically created, then optimized with base RHF/STO-3G [49], B3LYP/6-31+G** and B3LYP/6-311+G** [50]. Once the calculations were completed, a minimum energy structure was obtained in the ground state. The final molecular geometry of Aza was decorated with substituents of each ligand to obtain the structures of all eight C-seco limonoids. Then, they were optimized with base B3LYP/6-311+G** to obtain our eight 3D models, as outlined in the review published by Qin-Gang Tan et al. [11]. Furthermore, the structure of our reference ligand ecdysone was generated starting from its steroid scaffold, which was found in Gaussian 16 with its built-in parameter set. The structure was optimized with the base B3LYP/6-311+G** [12]. 

Due to the absence of a three-dimensional structure of the target biomolecule in the PDB database [13], a three-dimensional model of the ecdysone 20-monooxygenase enzyme was needed. To generate this 3D model of the hitherto unknown E20MO target by protein homology, we searched for related structural templates in the PDB database [13]. To this end, potential 3D templates were retrieved from www.rcsb.org (accessed on 19 November 2023). We evaluated the similarities between potential 3D templates, applying multiple sequence alignment techniques (MSA) under PSI-BLAST [15,51] against the PDB databank (www.rcsb.org, accessed on 19 November 2023). Details about the homology protein modeling technique have been described elsewhere [52]. 

The following evaluation criteria were considered: (i) overall identity between template and target sequences, (ii) homology of conserved amino acids between template and target sequences, (iii) similarities between observed ligands at the binding site, (iv) binding modes at the binding site, (v) biological activities, and (vi) evolutionary distance. To this end, the outcome was listed with structurally known liganded members of the mono-oxygenase protein. 

For the next step, the selected 3D template (PDB code 4ZGX [18]) contained the required heme group (see Figure 2). It was sent as input for the web-based 3D template modeling by Swiss-model (www.swissmodel.expasy.org, accessed on 19 November 2023) [29]. 

Finally, molecular docking simulations were performed between the nine limonoid-like ligands and the target proteins. To this end, the molecular interactions between the ligands and the modeled proteins were assessed under Autodock Tools using Autogrid4 and Autodock4 extensions [30,53]. The liganded complex of the selected 3D template was self-docked as a proof of concept, also known as the back docking test, with a known reference [53,54]. 

As an alternative target-free approach, we discarded the study by quantitative structure–activity relationships (QSARs). With only very few tested molecules in the compound series, a reliable QSAR model cannot be established concerning any beneficial or toxic agents [52]. To this regard, a promising computational approach was recently published describing a grid-based computational method, which was applied to eleven organophosphate compounds in use as agricultural pesticides [55].

## 5. Conclusions

The food and agrochemical industries have focused on essential oil limonoids. Our in silico study contributes with molecular mechanism insight through theoretical results corroborating the empirical findings. They describe the insecticidal activity of the ring C-type limonoids by inhibiting the hormone release upon binding at the active site of the reported target ecdysone 20-mono-oxygenase. Precisely, our ring C-type limonoids belong to the natural compounds of the formula type CxHyOz. While a larger portion of the ligand scaffold constitutes nonpolar aliphatic chains, the oxygen atoms and the aryl rings account for the negative charge density patches on the molecules’ surface, which lead to an attractive hydrogen-bonding network with binding-site residues. The target protein was generated by homology modeling, and the heme group merged into the cavity after superposition with the (heme-bearing) 3D template. Docking simulations revealed high target affinities of the limonoid insecticides, in close range to the insect hormone ecdysone and back docked ligand of the reference complex. The back docking validation test yielded not only the same pose but also the same affinity level as assessed by experimental data. With a validated procedure at hand, as a direct result, the strongest binding poses of all ligands were taken and their final docked poses compared after the superposition operation. Each ligand–receptor complex was analyzed for the interaction pattern and graphically documented (see Figures S1–S10 in Supplementary Information). All told, it seems not far-fetched to assume that our limonoids are strong binders to the E20MO enzyme as their biomolecular target for pesticide action. However, these findings have only a preliminary character since ecdysone 20-mono-oxygenase was never experimentally assessed as a target in the literature, which qualifies it as a mere putative target.

## Data Availability

The data presented in this study are available in article (or Supplementary Materials).

## Supplementary Materials

The following supporting information can be downloaded at: https://www.mdpi.com/article/10.3390/molecules29071628/s1; Figure S1: Azadirachtin A; Figure S2: Azadirachtin D; Figure S3: Azadirachtin G: Figure S4: Azadirachtin K: Figure S5: Cinnamoylmelianolone; Figure S6: Desepoxyazadirachtin A: Figure S7: Cinnamoy-Dihydroxymeliacarpin; Figure S8: Diacetyl-Deoxa-Oxomeliacarpin; Figure S9: Vepaol; Figure S10: Ecdysone. Table S1: Listing of target protein E20MO; Table S2: Listing of three hits among heme-containing enzymes; Table S3: Ligands chemically related to limonoids; Table S4: Reference molecule ecdysone and related PDB ligands; Table S5: Listing of maximum and minimum free energies from docking.
