# Supplementary material for: Computational Binding Study Hints at Ecdysone 20-Mono-Oxygenase as the Hitherto Unknown Target for Ring C-Seco Limonoid-Type Insecticides
Source: Molecules. 2024 Apr 5;29(7):1628. doi: 10.3390/molecules29071628 (PMC11013123; doi:10.3390/molecules29071628)
Supplement: Supplementary file 1 [file molecules-29-01628-s001.zip › molecules-2935772-supplementary.pdf]

Supplementary Information

Computational binding study hints at ecdysone 20-mono-oxygenase as the hitherto unknown target for ring C-seco limonoid-type insecticides

Ramsés E. Ramírez, Ricardo E. Buendia-Corona, Ivonne Pérez Xochipa, Thomas Scior

The protein sequence of the target enzyme E20MO was obtained from the Uniprot database (Q9VUF8) for the fruit fly (Drosophila melanogaster.

Table S1. Listing of target protein E20MO (first entry) and eight preselected PDB entries of liganded complexes as possible 3D templates for modeling of structurally unknown target E20MO sorted by % ID > ID. The selection criterion was the percentage identity score (% id) by MSA (Blast-P). In bold face appears the final selection (PDB entry **4ZGX**). Ligand QHC, short for: N - [(8R) -4- (4-chloro-3-fluorophenyl) -5,6,7,8-tetrahydroisoquinolin-8-yl] propanamide.

PDB ID	Organism	Sequence coverage %	% id	Ligand
Target E20MO (PDB entry does not exist)	Drosophila melanogaster (Fruit fly)	540 aa. Uniprot entry code: Q9VUF8	100%	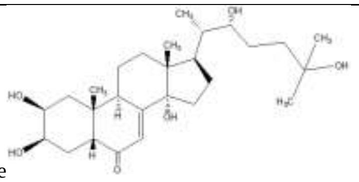 Ecdysone
4R20 CytochromeP450 17a [15]	*Danio rerio* (Zebra fish)	32%, 486 aa.	29%	Abiraterone 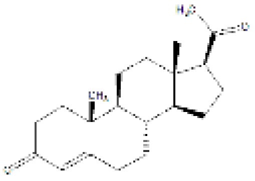
3CBD Octane monooxygenase [16]	*Bacillus megaterium*	40% 455 aa.	27%	N-palmitoglicina 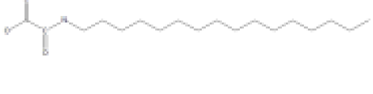
5VBU: CytochromeP450 21a2	*Homo sapiens*	31% 476 aa.	27%	Hydroxyprogesterone

[17]				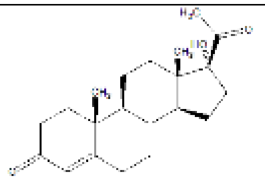
**4ZGX:** aldosteronesynthase [18]	*Homo sapiens*	68% 489 aa.	26%	QHC 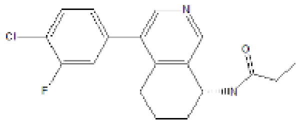
5OG9; WIFI-WC complex [19]	*Bacillus megaterium*	40% 473 aa.	26%	Testosterone 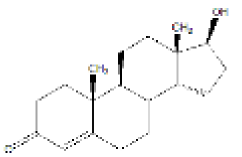
5EAF: CYP51 [20]	*Saccharo-myces cerevisiae*	42% 539 aa.	23%	Fluconazole 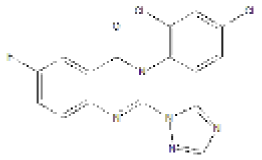
5JL9: CYP19A1 [21]	*Homo sapiens*	84% 503 aa.	21%	Androstenedione 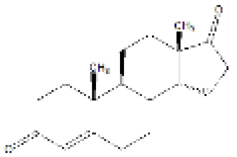
4UYL: essterol 14-alpha demethylase [22]	*Neosartorya fumigate*	43%, 470 aa.	21%	Voriconazole 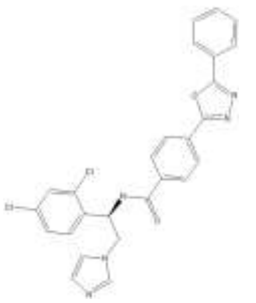

Table S2. Listing of three hits among heme-containing enzymes (CYPs) with structurally related ligands to ecdysone target ligand in the PDB database.

PDB ID	Organism	Sequence coverage, aa length	% id	Ligands
4GQS CYP 2C19 [23]	*Homo sapiens*	45%, 477aa.	26%	OXV 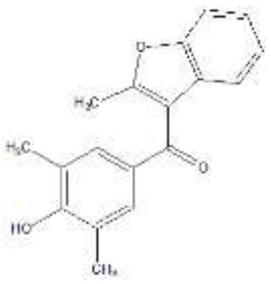
3G5N: cytochrome P450 2B4 [24]	*Oryctolagus cuniculus* (Rabbit)	45% 476 aa.	26%	1-(biphenyl-4-ylmethyl)-1H-imidazol 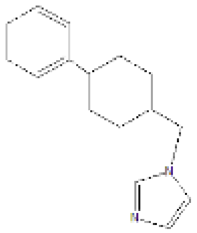
4NY4: CYP3A4 [25]	*Homo sapiens*	89% 484 aa.	23%	2QH 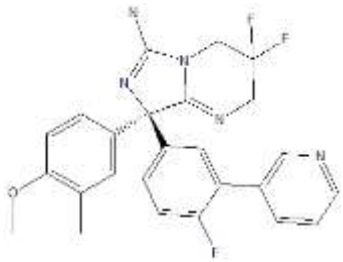

Table S3. Ligands chemically related to limonoids. Out of 841 PDB hits, seven structures were retained.

PDB ID	Ligand
5FOI [35] EC: 1.14	Micinamicin

	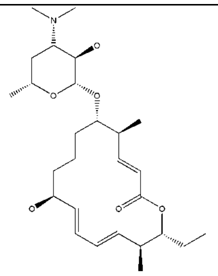
3SN5<br>[36]<br>EC: 1.14.14.23	colest-4-en-3-one<br>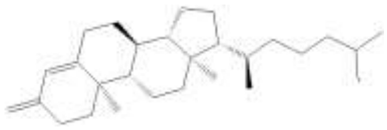
1DZ4<br>[37]<br>EC: 1.14.15.1	Camfor<br>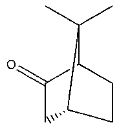
1T2B<br>[38]<br>EC: 1.14.14.133	Tropan<br>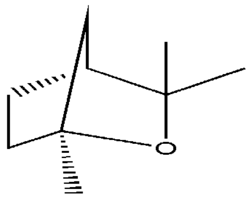
4DVQ<br>[39]<br>EC: 1.14.15.5	Desoxicorticosterona<br>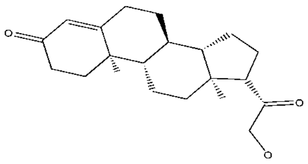
4WNU<br>[40]<br>EC: 1.14.14.1	Quinidina<br>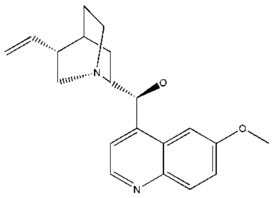
4WNV	Quinina

[40]<br><br>EC: 1.14.14.1	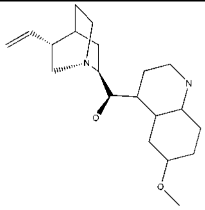

Table S4. Reference molecule ecdysone and related PDB ligands interacting at protein binding sites with heme group of enzymes of the CYP family (EC: 1.14).

Ligand	Structure	Features
Ecdysone	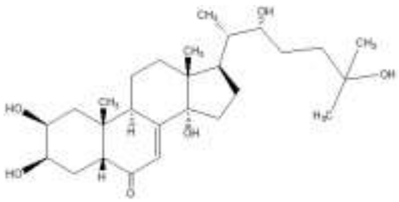	Formula: C27 H44 O6<br>Atoms: 77<br>Molecular weight: 464,635 Daltons<br>Total surface area (TSA): 726.3 Å² (ds = 15.2 Å)<br>Polar surface area (PSA): 227.3 Å² (apolar = 499.0 Å²)<br>Molecular volume: 457.2 Å³ (dv = 9.6 Å)<br>PSA / TSA: 0.3129<br>Rotatable bonds (ROBO): 10<br>Non rotatable bonds (NRB): 0<br>Aromatic carbons: 0<br>Rings: 4
Colest-4-en-3-one (PDB ID: 3SN5)	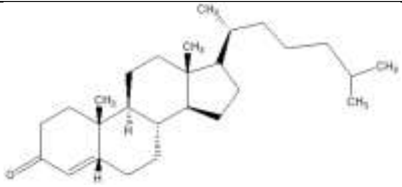	Formula: C27 H44 O<br>Atoms: 72<br>Molecular weight: 384,638 Daltons<br>TSA: 682.6 Å² (ds = 14.7 Å)<br>PSA: 45.6 Å² (apolar = 637.0 Å²)

		Molecular volume: 415.5 Å³ (dv = 9.3 Å)<br>PSA / TSA: 0.0668<br>ROBO: 5<br>NRB: 0<br>Aromatic carbons: 0<br>Rings: 4
Mycinamicin (PDB ID: 5FOI)	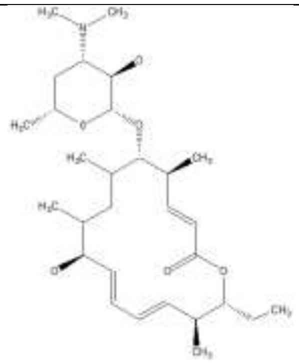	Formula: C29 H47 N O6<br>Atoms: 83<br>Molecular weight: 505,687 Daltons<br>TSA: 783.0 Å² (ds = 15.8 Å)<br>PSA: 93.0 Å² (apolar = 690.0 Å²)<br>Molecular volume: 499.8 Å³ (dv = 9.8 Å<br>PSA / TSA: 0.1188<br>ROBO: 4<br>NRB: 0<br>Aromatic carbons: 0<br>Rings: 2
Azadirachtin A (no PDB code, structure optimized)	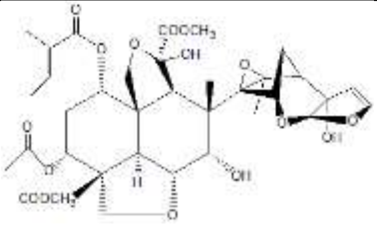	Formula: C35 H44 O16<br>Atoms: 95<br>Molecular weight: 720,714 Daltons<br>TSA: 861.1 Å² (ds = 16.6 Å)<br>PSA: 211.1 Å² (apolar = 650.0 Å²)<br>PSA / TSA: 0.245<br>ROBO: 13<br>NRB: 0<br>Aromatic carbons: 2

		Rings: 6
1-cinamoylmelianolone	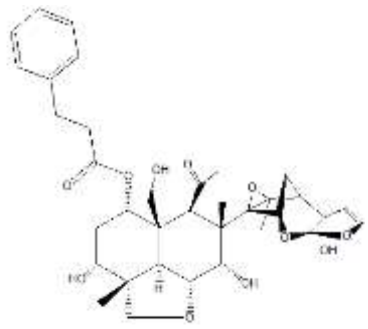	Formula: C35 H42 O11<br>Atoms: 88<br>Molecular weight: 638,701 Daltons<br>TSA: 832.3 Å^2 (ds = 16.3 Å)<br>PSA: 224.0 Å^2 (apolar = 608.3 Å^2)<br>Molecular volume: 576.6 Å^3 (dv = 10.3 Å)<br>PSA / TSA: 0.2691<br>ROBO: 11<br>NRB: 0<br>Aromatic carbons: 8<br>Rings: 6
QHC (PDB ID: 4ZGX)<br><br>This is the 3D template for target model E20MO4ZGX	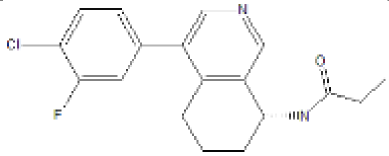	Formula: C18 H18 N2 O F Cl<br>Atoms: 41<br>Molecular weight: 332,800 Daltons<br>TSA: 544.7 Å^2 (ds = 13.2 Å)<br>PSA: 64.0 Å^2 (apolar = 480.7 Å^2)<br>Molecular volume: 287.5 Å^3 (dv = 8.2 Å)<br>PSA / TSA: 0.1175<br>ROBO: 3<br>NRB: 1<br>Aromatic carbons: 11<br>Rings: 3

Table S5. Listing of maximum and minimum free energies of binding to target model E20MO4ZGX. Asterisk symbols: * Reference ligand QHC was back docked against its crystal structure 4ZGX which was also the 3D template for target model generation by homology (**values in bold face**); ** Ligand QHC blind docked against 3D model of target E20MO4ZGX.

**Ligand**	**Free Energy of Binding (ΔG) [kcal/Mol]**	**Inhibition constant (Ki) [nM]**
I	-9	136
II	-10	37
III	-10	23
IV	-12.	4
V	-13	1
VI	-11	18
VII	-12	3
VIII	-11	19
IX	-9	473
Ecdisone (X)	-12	32
QHC *	**-11**	**4**
QHC **	-9	91

In **Figures S1 to S10** pairwise superpositions of the final docked poses for all nine limonoid ligands (magenta) and the reference ligand ecdysone (green) at the binding site of target Ecdysone 20-monoogygenase. Green tubes H-Bond, Yellow tubes π-π interactions, by molecular modeling software UCSF Chimera 09.

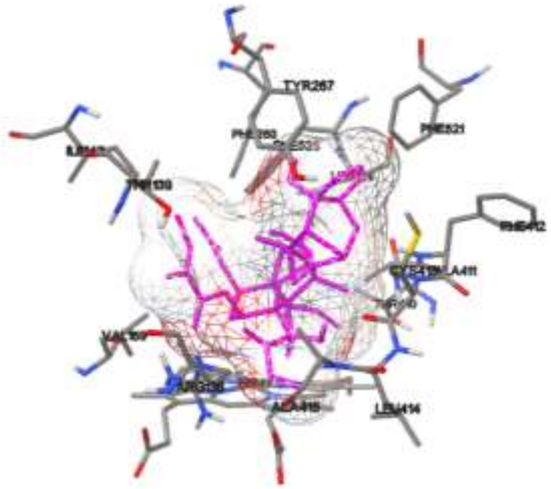

Figure S1. I Azadirachtin A.

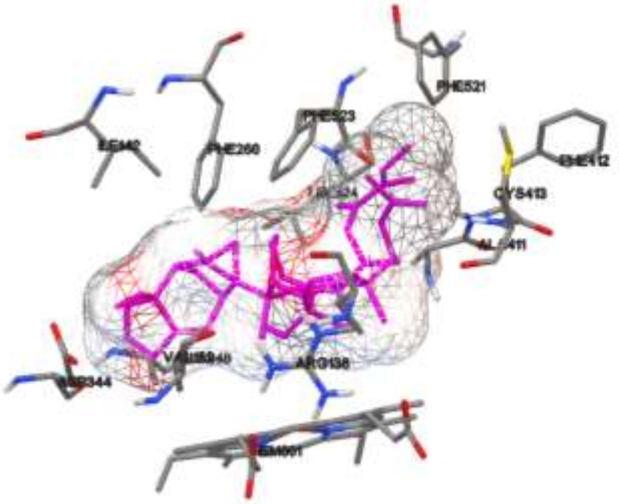

Figure S2. II Azadirachtin D

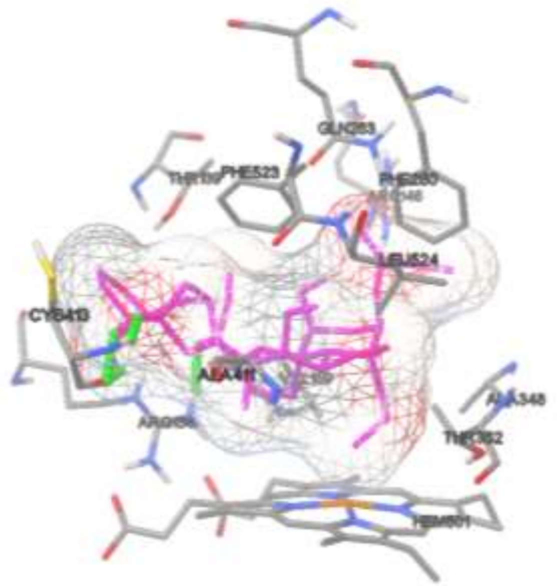

Figure S3. III Azadirachtin G

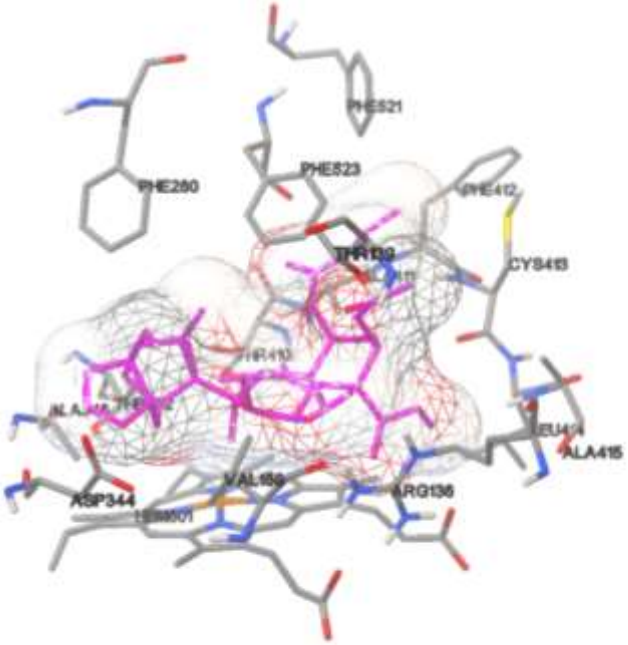

Figure S4. IV Azadirachtin K

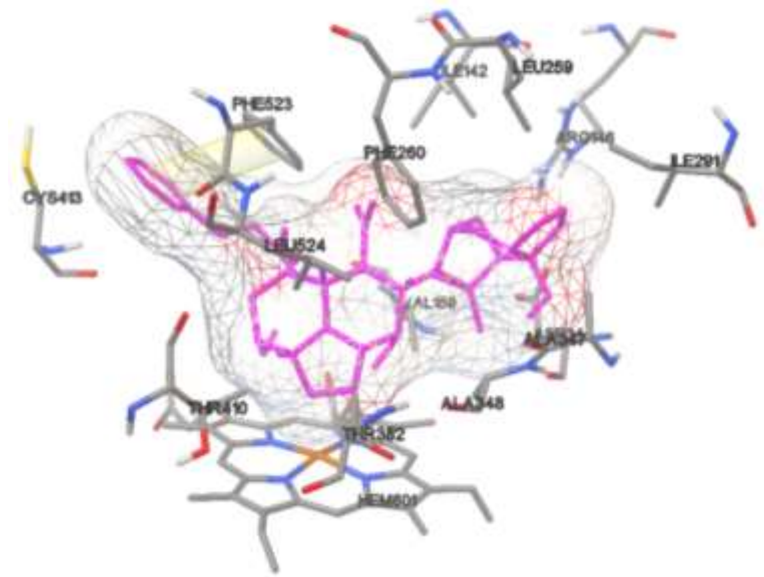

Figure S5. V 1-Cinnamoylmelianolone

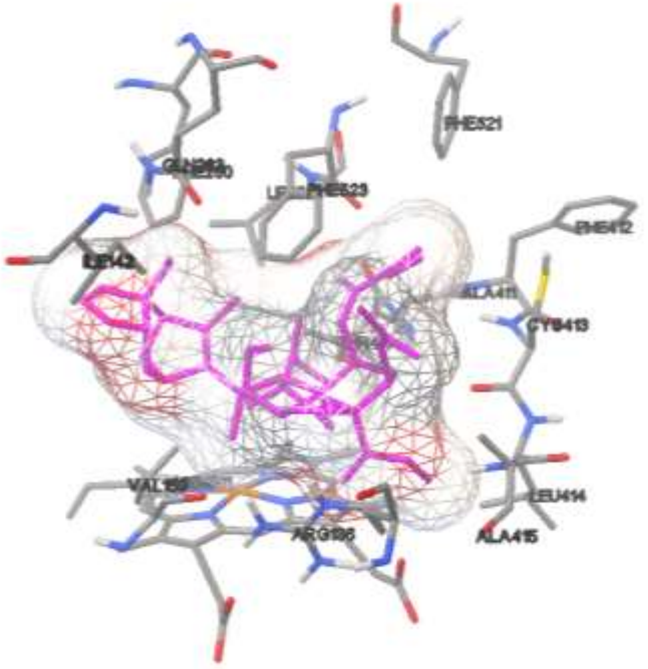

Figure S6. VI 13,14-Desepoxyazadirachtin A

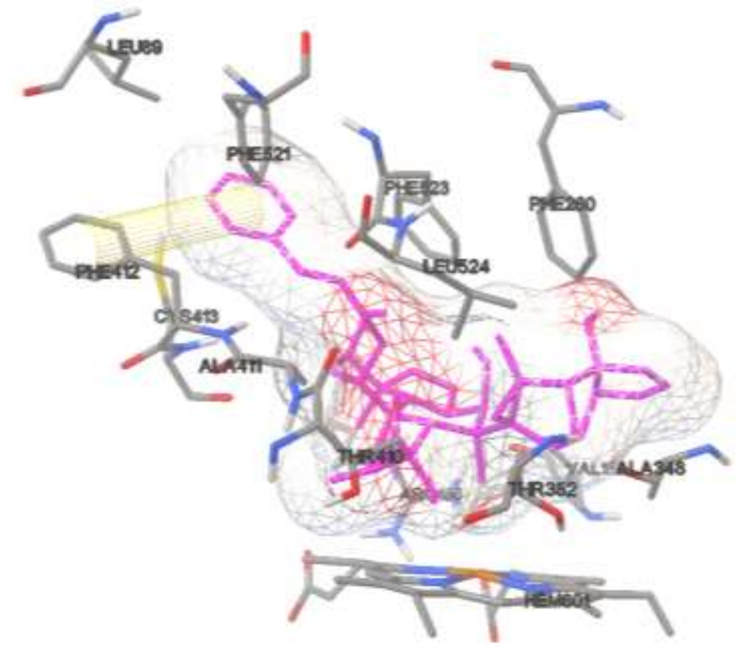

Figure S7. VII 1-Cinnamoyl-3,11-Dihydroxymeliacarpin

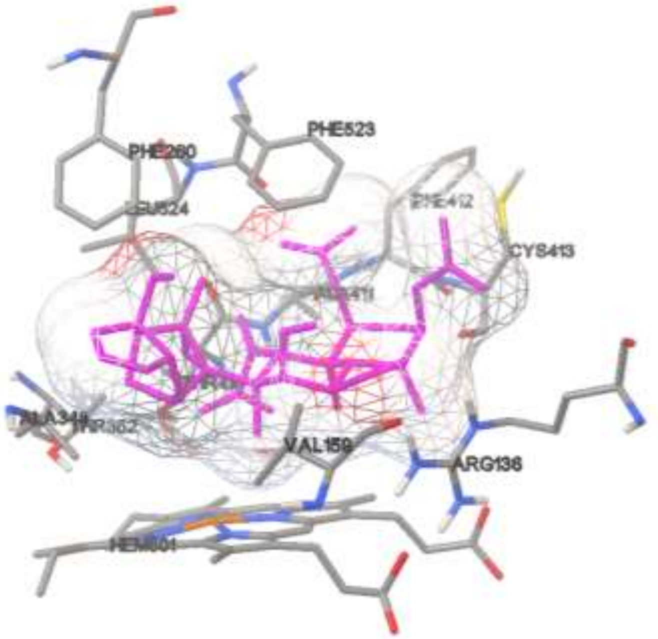

Figure S8. VIII 1,3,-Diacetyl-11,19-Deoxa-11-Oxomeliacarpin

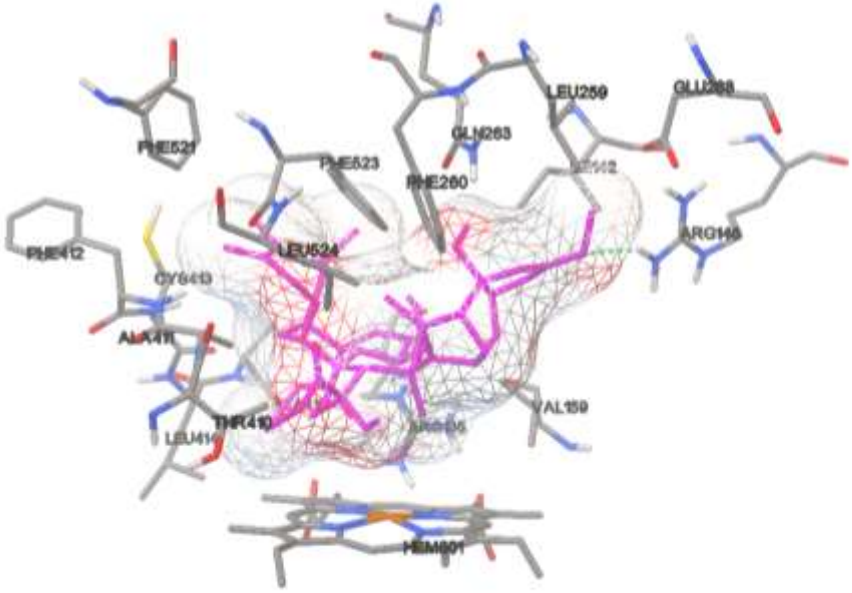

Figure S9. IX Vepaol

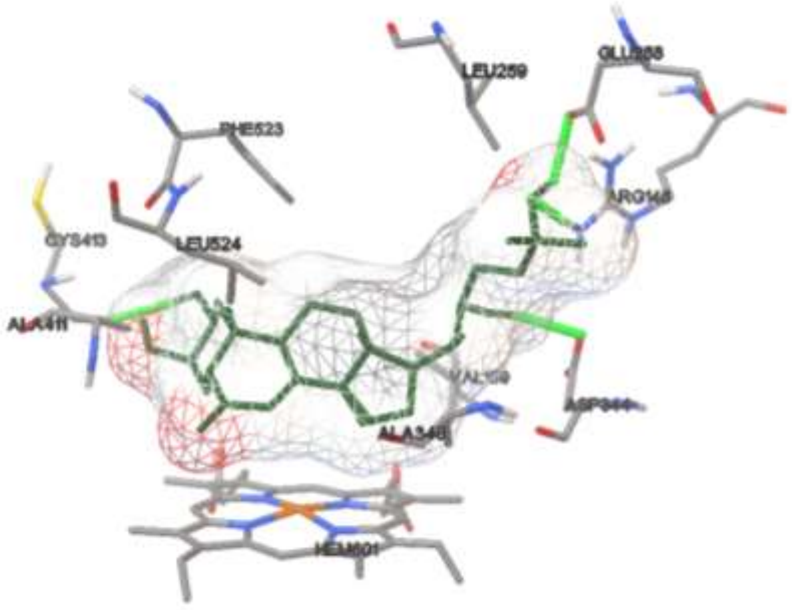

Figure S10. X Ecdysone
